# Validation of the Headache Impact Test (HIT-6™) across episodic and chronic migraine

**DOI:** 10.1177/0333102410379890

**Published:** 2011-02

**Authors:** Min Yang, Regina Rendas-Baum, Sepideh F Varon, Mark Kosinski

**Affiliations:** 1 QualityMetric, Inc., USA.; 2 Allergan, Inc., USA.

**Keywords:** HIT-6, migraine, chronic migraine, episodic migraine, headache

## Abstract

*Objective*: The purpose of this study was to assess psychometric properties of the six-item Headache Impact Text (HIT-6™) across episodic and chronic migraine.

*Methods:* Using a migraine screener and number of headache days per month (HDPM), participants from the National Survey of Headache Impact (NSHI) study and the HIT-6 validation study (HIT6-V) were selected for this study. Eligible participants were categorized into three groups: chronic migraine (CM: ≥ 15 HDPM); episodic migraine (EM: < 15 HDPM); non-migraine headaches. Reliability and validity of the HIT-6 were evaluated.

*Results:* A total of 2,049 survey participants met the inclusion/exclusion criteria for this study. Participants were identified as 6.4% CM; 42.1% EM; 51.5% non-migraine, with respective mean HIT-6 scores: 62.5 ± 7.8; 60.2 ± 6.8; and 49.1 ± 8.7. High reliability was demonstrated with internal consistency (time1/time2) of 0.83/0.87 in NSHI, and 0.82/0.92 in HIT6-V. Intra-class correlation for test-retest reliability was very good at 0.77. HIT-6 scores correlated significantly (*p* < .0001) with total Migraine Disability Assessment Scale scores (*r* = 0.56), headache pain severity (*r* = 0.46), and HDPM (*r* = 0.29). Discriminant validity analysis showed significantly different HIT-6 scores (*F* = 488.02, *p* < .0001) across the groups.

*Conclusion*: Results from these analyses confirm that the HIT-6 is a reliable and valid tool for discriminating headache impact across episodic and chronic migraine.

## Introduction

Migraine is a prevalent and disabling condition that affects approximately 12% of the population in the Western countries (1,2). The detrimental effects of migraine are well documented. Migraine interferes with an individual’s physical functioning, work productivity, leisure activities, lifestyle and psychological well-being (3–7). Furthermore, research has shown that the level of disability differs according to frequency of headache events. According to Bigal and Lipton (8–10), many patients with migraine can experience the disease progression clinically, physiologically, and anatomically. The typical clinical progression of migraine is defined by the frequency of headache days. Individuals with episodic migraine (EM), defined as having <15 headache days per month (HDPM), may progress to chronic migraine (CM), defined as ≥15 HDPM (8–10). While the pathophysiology of the transformation from episodic to chronic migraine is not well understood, CM has been shown to be significantly more debilitating than episodic migraine (3,6,11).

Regardless of its disruptive effect, migraine is frequently underdiagnosed and undertreated (2,6,12). A potential reason, as proposed by Bigal et al. (6), is the lack of physician confidence in the diagnosis of chronic headache conditions and their subtypes. The International Classification of Headache Disorders, second edition (ICHD-II) (13), introduced diagnostic criteria for CM, a complication of migraine. Subsequently, the criteria were refined and revised to more accurately reflect the large majority of CM patients seen in clinical practice (14).

Outcome measures to capture the impact of the disorder, beyond the frequency of headache episodes or headache days, have been recommended and used by clinical experts (15–18). These measures have most commonly been used in studies of acute and prophylactic treatments for episodic migraine. Few trials have reported evaluation of treatments for CM (14). Clinical evaluation of migraine-related disability or quality of life plays an important role in migraine research and patient management in clinical practice. To our knowledge, none of the measures that evaluate disability or quality of life have been psychometrically validated for differentiating between the persons with EM disorder (<15 HDPM) and CM disorder (≥15 HDPM).

The six-item Headache Impact Test (HIT-6) was designed to provide a global measure of adverse headache impact (19,20) and was developed to use in screening and monitoring patients with headaches in both clinical practice and clinical research (20). The HIT-6 items measure the adverse impact of headache on social functioning, role functioning, vitality, cognitive functioning and psychological distress. The HIT-6 also measures the severity of headache pain. The six items were selected from 89 items (54 from an existing adverse headache impact item pool and 35 items recommended by clinicians) (20). The HIT-6 shows good internal consistency and test-retest reliability, construct validity and responsiveness in general headache patients (19–21). Since its initial development and validation, the HIT-6 has been well received and widely utilized in clinical practice, and applied to clinical trials for patient screening and treatment monitoring of headaches, including migraine (22–28).

The HIT-6 was developed among headache sufferers with different headache day frequency and severity levels. Given the increasing use of the HIT-6 in clinical management of patients, as well as in clinical research, it is necessary to provide evidence for the reliability and validity of the HIT-6 in migraine patients who suffer varying headache day frequency. The purpose of this study was to evaluate the reliability and validity of the HIT-6 among migraine patients, and its ability in discriminating headache impact and the level of disability between EM and CM disorders.

## Methods

### Data sources

Data came from two sources of adult participants with recent headache complaints: (i) the National Survey of Headache Impact (NSHI) study (19) and (ii) the HIT-6 Validation (HIT6-V) study (20). The NSHI study was a 1999 telephone interview survey study using a randomly generated list of household telephone numbers from 48 states of the USA. Eligible participants were: (i) 18 to 65 years of age; (ii) permanent US residents; (iii) those with at least one headache in the four weeks prior to the interview that was not related to a cold, flu, a head injury or a hangover; and (iv) able to converse easily in English. Follow-up interviews after three months were completed for a subgroup of respondents randomly selected from the initial respondents to represent mild, moderate and severe headache sufferers. The severity levels associated with pain from headaches in general were derived based on a numeric rating scale of headache pain from 0 to 10, 0 representing “no pain” and 10 representing “pain as bad as it can be.” Mild headache pain was defined by scores 1–4; moderate by scores 5–7; and severe by scores 8–10. The purpose of the NSHI study was to obtain empirical data for the development of the Headache Impact Test item pool using 54 items from four widely used measures of headache impact, along with 35 new experimental items proposed by clinicians (19,21). The four headache impact measures were administered in the following order: (i) the Migraine Disability Assessment (MIDAS) score; (ii) the Migraine-Specific Quality of Life (MSQ) questionnaire; (iii) the Headache Impact Questionnaire (HIMQ); and (iv) the Headache Disability Inventory (HDI). Details of the NSHI have been described elsewhere (19).

Data of the HIT6-V study were collected in 2000 through the America Online (AOL) Opinion Place from AOL subscribers who were (i) 18 to 65 years of age, (ii) with at least one headache in the past four weeks not related to a cold, flu, a head injury or a hangover; and (iii) agreed to be contacted again in two weeks to complete the follow-up survey. At time 1, the HIT-6 was administered first followed by the MIDAS and the QualityMetric's Short-Form (SF)-8 Health Survey (which measures eight health domains). At time 2, this same order was used except that 3 items which assessed changes in headache impact since time 1 were added following the MIDAS items and preceding the SF-8. The purpose of this study was to test the validity of the HIT-6 in headache suffers. Detailed study design of the HIT6-V has been described elsewhere (20).

In both studies, data were collected on headache participants. Given the similarities in the inclusion and exclusion criteria of the two studies and in order to ensure an adequate sample size in the EM and CM groups, data from the two studies were pooled when applicable. For instance, data on the HIT-6 and MIDAS were collected in both studies, and thus pooled.

The final HIT-6 score is obtained from simple summation of the six items and ranges between 36 and 78, with larger scores reflecting greater impact. Headache impact severity level can be categorized using score ranges based on the HIT-6 interpretation guide (29), The four headache impact severity categories are little or no impact (49 or less), some impact (50–55), substantial impact (56–59), and severe impact (60–78). See Appendix A for the HIT-6 questionnaire.

MIDAS was developed to assess headache-related disability of migraine patients (30). MIDAS included five questions, capturing information on missed days of work, household chores, non-work activity and days with substantially reduced productivity over a three-month period. A total score is calculated by adding the five headache-related disability items together. Higher scores indicate increased disability due to headache. The total MIDAS score can be further used to define four grades of headache-related disability, including grade I for “minimal or infrequent disability” (0–5); grade II for “mild or infrequent disability” (6–10); grade III for “moderate disability” (11–20); and grade IV for “severe disability” (21+) (30). The grades were determined based on physicians’ judgment of MIDAS scores on varying levels of migraine patient’s activity limitation and treatment needs (30). See Appendix B for the MIDAS questionnaire.

The SF-8 Health Survey is a brief generic health survey for measuring health-related quality of life (HRQOL) (31). The SF-8 includes eight questions representing the same eight health domains produced by the SF-36 Health Survey, including physical functioning (PF), role limitations due to physical health (RP), bodily pain (BP), general health (GH), vitality (VT), social functioning (SF), role limitations due to emotional problems (RE) and mental health (MH). Weighted scores for these domains can be aggregated into the physical component summary (PCS) and mental component summary (MCS) measure scores. Scoring of the SF-8 used a norm-based approach with scores centered around 50 reflecting the average domain scores of the general US population (31). Higher scores indicate better health. Data on the SF-8 Health Survey were collected in the HIT6-V study only.

### Migraine group classifications

The ID Migraine™ is an epidemiological migraine screener that has been reported to be reliable, valid, and efficient for identifying migraine patients in primary care (32). Only participants who had values on ID Migraine and provided the number of headache days they experienced over the last three months were included in the current study. Participants were considered as migraine positive if they met the ID Migraine criteria. Participants identified with migraine were further grouped based on HDPM (8–10). Those patients identified as migraine positive who reported having ≥15 HDPM were considered to have CM. Those identified as migraine positive who responded having <15 HDPM were considered to have EM. The remaining study participants were classified as non-migraine headache participants.

### Statistical analysis

Internal consistency reliability of the HIT-6 among migraine participants was assessed by evaluating Cronbach’s α for the HIT-6 scores across the two studies and by the pooled sample. A Cronbach’s α value ≥0.8 is considered as good internal reliability (33). Intra-class correlation (ICC) was examined for test-retest reliability. Because the time interval between time 1 and time 2 was two weeks in the HIT6-V study compared to three months in the NSHI study, test-retest reliability can only be estimated in the HIT6-V study. ICC values of at least 0.5 were considered indicative of fair test-retest reliability (34).

Construct validity was assessed through an examination of the Spearman correlation coefficients between HIT-6 scores and the SF-8 scores (domains and summary measures), as well as with the total MIDAS score, headache pain severity (0–10 scale) and number of HDPM. We hypothesized that at least small to moderate correlations were present between the HIT-6 scores and the specified variables. Assessment of the instrument’s discriminant validity was based on the method of known-groups validity (35). Known-groups validity was assessed using analysis of variance (ANOVA) methods comparing mean HIT-6 score differences across CM, EM and non-migraine headache participants. We expected that higher mean HIT-6 scores would be observed in the more disabled groups. In addition, the HIT-6 scores were grouped into four impact severity categories using the interpretation guide in the HIT-6 manual (29). Given the directional nature of the impact severity levels and the headache staging (i.e. CM, EM, and non-migraine headache), we hypothesized that non-migraineurs, those with EM and those with CM would, in that order, have increasing impact severity level defined by the HIT-6 scores. The proportional odds model was used to evaluate the hypothesized association. Model adequacy was assessed using test of proportional odds assumption (36). Other than discriminant validity tests, all psychometric property assessments were conducted among the migraine participants (both EM and CM together).

## Results

A total of 2049 participants were included in the current study, of which 1096 (53.5%) were from the NSHI study. A total of 994 participants (48.5% of the 2049 participants) were identified as having migraine, of which 624 (62.8%) were from the NSHI study. The majority of the participants were female (75.0%) and slightly over half (56.3%) were between 18 and 39 years of age. Based on the classification criteria defined in the "Methods" section above, 6.4% of the study participants had CM (*N* = 131) and 42.1% had EM (*N* = 863). The rest of participants (51.5%) had non-migraine headaches (*N* = 1,055). Participant characteristics are presented in Table 1. Approximately half of the participants in the NSHI and 60% of the participants in the HIT6-V were younger than 40 years old. Approximately three-quarters (72.8% in NSHI and 77.4% in HIT6-V) were females.

**Table 1.** Participants' characteristics and comparisons by episodic and chronic migraine status and study

	Non-migraine		Episodic migraine		Chronic migraine	
	NSHI *N* (%)	HIT6-V *N* (%)	NSHI *N* (%)	HIT6-V *N* (%)	NSHI *N* (%)	HIT6-V *N* (%)
Age groups*						
18–29	114 (24.1)	116 (21.9)	168 (30.4)	80 (27.8)	23 (32.4)	11 (20.8)
30–39	157 (33.3)	126 (23.7)	199 (36.0)	72 (25.0)	25 (35.2)	17 (32.1)
40–49	134 (28.4)	152 (28.6)	128 (23.1)	70 (24.3)	19 (15.3)	19 (35.8)
50–69	67 (14.2)	137 (25.8)	58 (10.5)	66 (22.9)	4 (5.6)	6 (11.3)
Gender^†^						
Female	298 (63.1)	421 (72.5)	435 (78.7)	269 (87.1)	65 (91.5)	45 (76.3)
Male	174 (36.9)	160 (27.5)	118 (21.3)	40 (12.9)	6 (8.5)	14 (23.7)

HIT6-V: HIT-6 Validation Study. NSHI: National Survey of Headache Impact.

* 81 participants had missing value on age.

† 4 participants had missing value on gender.

### Reliability analyses

Results of reliability analyses of the HIT-6 among the migraine sufferers are presented in Table 2. The time interval between the two assessments (time 1 and time 2) was different for the NSHI study and the HIT6-V study (three months vs. two weeks, respectively). The internal consistency reliability (Cronbach’s α) of the HIT-6 was 0.83 at time 1 and 0.87 at time 2 for the NISH study and was 0.82 at time 1 and 0.90 at time 2 for the HIT6-V study. The internal consistency reliability of the pooled migraine sample was 0.83 at time 1 and 0.90 at time 2. The intra-class correlation coefficient for test–retest reliability between the HIT-6 scores at time 1 and time 2 for the HIT6-V study was 0.77.

**Table 2.** Reliability estimates for HIT-6 among migraine participants

Reliability test	Coefficient
Internal consistency reliability (Cronbach’s α) – NSHI^a^	
Time 1	0.83
Time 2	0.87
Internal consistency reliability (Cronbach’s α) – HIT6-V^b^	
Time 1	0.82
Time 2	0.90
Internal consistency reliability (Cronbach’s α) – total sample	
Time 1	0.83
Time 2	0.90
Test-retest scale reliability – HIT6-V^b^	0.77

HIT6-V: HIT-6 Validation Study. NSHI: National Survey of Headache Impact.

a Time interval between time 1 and time 2 of the NSHI study was 3 months.

b Time interval between time 1 and time 2 of the HIT6-Validation study was 2 weeks.

### Validity analyses

Correlations between the HIT-6 scores and the total MIDAS scores, headache pain severity, number of HDPM, SF-8 scales and the summary measures are presented in Table 3. The HIT-6 scores significantly correlated with all of the criteria measures (*p* < 0.0001) with a small to moderate magnitude, supporting convergent validity. The highest correlation was observed between the HIT-6 scores and the total MIDAS scores (*r* = 0.56) HIT-6 scores and headache pain intensity were also moderately associated (*r* = 0.46), while a lower correlation with the number of HDPM was observed (*r* = 0.29).

**Table 3.** Correlations between HIT-6 scores and migraine criteria measures in migraine participants

Criterion Measure	Coefficient (*r*)
Headache days per month; *N* = 988)	0.29
Headache pain severity (0–10: *N* = 986)	0.46
MIDAS total score (*N* = 926)	0.260
SF-8 Scales^a^ (*N* = 624)	
Physical functioning	−0.24
Role physical	−0.29
Bodily pain	−0.18
General health	−0.20
Vitality	−0.17
Social functioning	−0.28
Role emotional	−0.24
Mental health	−0.16
SF-8 summary measures^a^ (*N* = 624)	
Physical component summary (PCS-8)	−0.26
Mental component summary (MCS-8)	−0.18

HIT-6 = 6-item Headache Impact Test. MIDAS = Migraine Disability Assessment scale.

All Spearman correlation coefficients were significant at <0.0001.

a SF-8 data were only collected in the HIT6-Validation study.

All correlations with SF-8 scales and summary measures were negative, as expected. The highest correlations were observed between the HIT-6 and the SF-8 role physical (RP) and social functioning (SF) scales and the lowest correlations were observed between the HIT-6 and the SF-8 vitality (VT) and mental health (MH) scales. In relation to the physical and mental health summary measures, a higher correlation was observed with the physical component summary (PCS-8) than the mental component summary (MCS-8).

In Table 4, the means and standard deviations of the scores on HIT-6, HDPM, headache pain severity, MIDAS, PCS-8 and MCS-8 are presented across the headache groups and the overall study sample. Results showed that the more severe the headache status, the worse the values on all of these scales. The mean HIT-6 scores were significantly different between the CM and EM sufferers (62.5 ± 7.8 vs. 60.2 ± 7.8, *p* = .0032). In discriminant validity analysis using groups known to differ in migraine diagnosis and headache frequency as the criterion measure (i.e. CE, EM and non-migraine headache sufferers), the HIT-6 showed large and statistically significant differences in mean scores across the diagnostic groups (*F* = 488.02, *p* < .0001).

**Table 4.** Means and standard deviations of the headache measures by groups

	Non-migraine	EM	CM	Total sample
*N*, %	1055, 51.5%	863, 42.1%	131, 6.4%	2,049, 100%
HIT-6*,^±^	49.1 ± 8.7	60.2 ± 7.8	62.5 ± 7.8	54.6 ± 10.1
HDPM*,^±^	3.7 ± 4.6	4.7 ± 3.3	21.4 ± 5.8	5.3 ± 6.0
Headache pain severity	4.6 ± 1.9	6.7 ± 1.8	7.1 ± 1.7	5.6 ± 2.2
MIDAS*,^±^	5.6 ± 9.5	21.0 ± 24.8	52.6 ± 46.5	14.7 ± 23.77
PCS-8*,^±^	50.4 ± 7.9	46.7 ± 8.9	42.6 ± 10.3	48.0 ± 8.9
MCS-8*,^±^	47.7 ± 10.0	44.3 ± 10.6	38.8 ± 11.2	45.5 ± 10.7

CM = chronic migraine. EM = episodic migraine. HDPM = headache days per month. MIDAS = Migraine Disability [assessment scale]. PCS-8 = physical component summary [SF-8 Health Survey]. MCS-8 = physical component summary [SF-8].

* Means were statistically significantly different (*p* < 0.01) across non-migraine headache, EM and CM groups.

± Means were statistically significantly different (*p* < 0.01) across EM and CM groups.

Table 5 shows the frequency and percentage of HIT-6 impact severity level by CM, EM and non-migraine headache. Based on the four impact categories derived from HIT-6 scores, for slightly over half (51.6%) of the non-migraine participants headache had little or no adverse impact to their daily life whereas very few in either migraine where in this impact category (EM = 10.9%; CM = 7.8%). Over a half of the EM participants and nearly three-quarters in the CM groups indicated that their headache had severe adverse impact on their daily life. In contrast, only 15.2% of the non-migraine headache participants indicated that their headache had severe adverse impact to their daily life. The proportional odds assumption was met (*X^2^*^ ^= 0.3834, *p* = . 9838), validating the use of the proportional odds model to describe the relationship between frequency based headache groups and HIT-6 headache impact levels. The model revealed that when compared to the non-migraine headache sufferers, the odds of reporting one level higher on the HIT-6 impact severity level were approximately eight times greater for EM (odds ratio [OR] = 8.6, 95% confidence interval [CI]: 7.3–10.7); and 13 times greater for CM (OR = 13.4, 95% CI: 9.0–19.9).

**Table 5.** Frequencies and percentages of headache staging by HIT-6 Impact severity level*

HIT-6 severity level	Non-migraine	EM	CM
Little or no impact	541 (51.6)	94 (10.9)	10 (7.8)
Some impact	232 (22.1)	109 (12.7)	12 (9.4)
Substantial impact	117 (11.2)	133 (15.5)	16 (12.5)
Severe impact	158 (15.2)	524 (60.9)	90 (70.3)
Total *N* (%)	1,048 (100)	860 (100)	128 (100)

HIT-6 = 6-item Headache Impact Test. CM = chronic migraine. EM = episodic migraine.

Little or no impact = HIT-6 score 49 or less. Some impact = HIT-6 score 50–55. Substantial impact = HIT-6 score 56–59. Severe impact = HIT-6 score ≥ 60.

13 patients with missing value on HIT-6 were excluded in this table.

* Study participants were significantly differently distributed across HIT-6 severity levels (X^2 ^= 607.31, *p* < .0001)

## Discussion

The clinical community is increasingly interested in improving the diagnosis and treatment paradigm for patients with chronic migraine. Although chronic migraine sufferers represent a small subgroup of the overall migraine population, the annual costs associated with CM patients (including both direct and indirect costs) are four times more than those with EM on a per-patient basis (37). Controlled clinical data on acute and prophylactic treatment of chronic migraine is limited (13,23) and consequently, there is little evidence-based medicine available to help physicians care for these patients. Recently introduced diagnostic criteria for CM should improve clinicians’ abilities to diagnose this highly disabling disorder. It is also necessary to ensure that valid and reliable measures are available to aid in the clinical evaluation of treatment. The HIT-6 questionnaire is a simple, easy to administer assessment that can be used as a clinical evaluation of the impact of headache on a patient’s quality of life. Because the HIT-6 was initially developed and validated in a broad range of headache suffers, it is important to establish its reliability and validity of the measure among migraine patients and to determine its ability to discriminate headache impact and level of disability between EM and CM.

In this study, the HIT-6 showed high internal consistency reliability among migraine sufferers, varying between 0.82 and 0.90. This is comparable to the original development study (0.84–0.90) among the general headache sufferers (20) and to the validation study among patients in a headache-specialty practice (0.87) (38). Our study also had similar test-retest reliability (0.77) to other studies which reported values between 0.77and 0.80 (20,38). Construct validity was supported by the convergent validity in HIT-6 correlation with the MIDAS total score headache pain intensity, the number of HDPM and the negative correlations with SF-8 scales and summary scores, as well as by the discriminant validity across the different stages of headache. The difference in mean HIT-6 score between chronic and episodic migraine was smaller (2.3) than the difference in mean MIDAS scores (21.6). Although the difference provided by MIDAS scores was nearly 10 times greater than that observed with the HIT-6, the two instruments do not share the same scale and thus these values cannot be directly compared. When taking into account the actual scale ranges, we find that the difference in mean HIT-6 scores between EM and CM patients is equivalent to roughly 5.5% of the scale range (2.3/[78–36]), whereas the difference obtained with the MIDAS is roughly equivalent to 11.7% (21.6/[270–0]). We note also that the minimally important difference of the HIT-6 in patients with chronic daily headache was estimated to be between −2.7 and −2.3 (39). Although not offering a direct comparison to the result observed in the current study, this range may provide an additional frame of reference. It is important to note that while the MIDAS questionnaire and the HIT-6 are both patient-reported measures, the constructs assessed by the respective instruments differ. The MIDAS asks sufferers to report the number of missed days of paid work, household chores and non-work activity as well as reduced productivity related to headaches, whereas the HIT-6 assesses the extent of headache impact on various aspects of daily life on a five-point scale ranging from never to always. Given that the MIDAS content focuses on lost time due to headaches and the HIT-6 measures impact of headaches, it is not unexpected that differences between EM and CM patients were greater for MIDAS than for HIT-6 scores. Both instruments assess important but different aspects of headache-related disability using scales that differ substantially. While each of the items in the MIDAS provides a more objective evaluation by asking about frequency of days affected by headache, the items in the HIT-6 may better reflect patients own evaluation of how headaches affect their life. This subjective evaluation of disease impact is, to a large extent, one of the key properties of patient reported outcomes (PROs), which aim to capture the impact of a disease on patients’ quality of life. Moreover, a one-month recall of the HIT-6 may offer a more accurate evaluation of impact than the three-month recall used in the MIDAS.

A small correlation coefficient between the HIT-6 and the number of HDPM was also expected. While it is important clinically to know the number of headache days, such a value only provides headache day frequency, but it does not reflect to what extent headache affects the sufferer’s daily life. Our findings regarding the relative strength of the association between HIT-6 scores and headache pain intensity and headache frequency agree with those of another study (40) that also reported HIT-6 scores to be more strongly associated with headache pain severity than with headache frequency. However, in another study (41) headache frequency, but not headache severity, was strongly related to HIT-6 scores. In this latter study, pain severity was measured through headache diaries, whereas our study and the study of Sauro (40) assessed pain severity through a single question. This important methodological difference may account for the disparity in findings. Nevertheless, despite differences in study measures used to collect headache characteristics and headache disability, other studies (42,43) have also reported that disability was more strongly associated with headache pain severity than with headache frequency.

Small negative correlations were observed between the HIT-6 and the SF-8 scales and summary scores. The magnitude of these correlations was smaller than the correlations from another study between the HIT-6 and the SF-36 Health Survey (38). In that study (38), the negative correlation coefficients varied between 0.22 and 0.57. The highest correlation was observed on the social functioning (SF) scale (*r* = −0.57), followed by the role physical (RP) limitation (*r* = −0.52). The weakest association was observed on the mental health (MH) scales (*r* = −0.22). While our study had much smaller correlation coefficients, a similar order of the magnitude of the correlations between the HIT-6 and the SF scale scores were observed where the strongest association was with RP and SF scales and the weakest was with the MH scale. The differences in the magnitude of the association between the HIT-6 and the SF-8 versus HIT-6 and the SF-36 are likely due to having more questions asked in the SF-36. Although, because it has more questions in each scale, the SF-36 provides more stable scale scores, additional questions may also capture aspects of headache impact that were missed in the SF-8.

Discriminant validity test showed that the HIT-6 scores differed significantly across the groups between CM, EM and non-migraine headache sufferers. When using the HIT-6 interpretation guideline to categorize the headache impact severity levels, our findings suggest that CM sufferers were more likely to report substantial or severe headache impact compared to EM or non-migraine sufferers. However, in a French study of the HIT-6 (44) similar HIT-6 scores were observed in chronic (≥15 days; *N* = 68) and episodic headache patients (<15 days; *N* = 75) who were seen in a migraine clinic (63.1 [time 1] and 62.5 [time 2] for chronic headache patients vs. 64.9 [time 1] and 62.9 [time 2] for episodic headache patients at time 1 and time 2, respectively). Differing findings between studies are likely due to the smaller size and possibly greater homogeneity of the sample in the French study.

The findings from our study have important clinical implications. As suggested by clinical experts, migraine is a chronic disorder that in some patients can be characterized as a clinically progressive disorder with increasing headache frequency (8,9,45). Bigal and Lipton (8,9) have suggested that migraine chronification should look beyond the clinical progression defined by attack frequency and also take into account the physiological and anatomical progression of the disorder. Given that those patients with CM have a much greater burden of disease and poorer quality of life (3–7), headache experts suggest that aggressive treatment should be provided to migraine patients in order to possibly prevent progression of the disorder and undo patient suffering (10).

A limitation of the study was that the migraine diagnosis was not based on physician report, but rather the participants’ self-report to a validated migraine screening tool, the ID Migraine Screener (32), was used. It is possible that some sufferers were misclassified as with or without migraine. Although the ID Migraine has demonstrated excellent accuracy properties (32), it is possible that respondents were misclassified relative to IHS diagnostic criteria. The results presented in its validation study, suggested that, the ID Migraine Screener had a slightly higher misclassification (specificity = 0.75) among patients who were classified by the ICHD-II criteria as not having migraine than among patients who met ICHD-II criteria for migraine (sensitivity = 0.81). These results suggest that overall our study could have been overly inclusive with respect to its identification of migraine cases. On the other hand, the use of ID Migraine Screener could have influenced, to some extent, the large differences in HIT-6 scores observed between non-migraine and migraine groups since the presence of disability (interference with activities) is one of the three items used in the ID Migraine Screener. Nevertheless, the development study of the ID Migraine Screener did select these 3 items from a larger pool of 9, suggesting that this particular set of items produced the best agreement with IHS criteria. Overall, we feel confident that the potential bias caused by the use of the ID Migraine Screener was, at most, small. Another limitation is that the study datasets came from two sources (i.e. the NSHI and HIT6-V). Due to somewhat different designs, some study analyses could only be performed using one dataset, but not the other. For instance, we were able to obtain a satisfactory result for test-retest analysis using the HIT6-V but not with NSHI due to lack of availability of such data for the test. In addition, for some tests we had a larger dataset to analyze because data from both sources were able to be pooled for analysis.

## Conclusion

Our study shows that the HIT-6 is a reliable and valid tool for measuring the impact of headache on daily life in both episodic and chronic migraine sufferers. Furthermore, the HIT-6 tool discriminates well between chronic migraine, episodic migraine and non-migraine patients. As a brief tool, the HIT-6 is easy to score and interpret, and can be readily integrated into clinical practice, or clinical studies of migraine patients. It may offer clinicians a practical and easy-to-implement tool to assist them with evaluating treatment effectiveness by obtaining input directly from the patient on aspects other than just the frequency of headache days.

## Appendix A

The HIT-6 is a copyright of Quality Metric Incorporated.

## Appendix B: The Migraine Disability Assessment (MIDAS) Questionnaire

With reprint permission from CVS Caremark for use as an Appendix in this paper
